# Development of functional noodles by encapsulating mango peel powder as a source of bioactive compounds

**DOI:** 10.1016/j.heliyon.2024.e24061

**Published:** 2024-01-04

**Authors:** Md Raihan Kabir, S.M. Kamrul Hasan, Md Rakibul Islam, Maruf Ahmed

**Affiliations:** Department of Food Processing and Preservation, Hajee Mohammad Danesh Science and Technology University (HSTU), Dinajpur-5200, Bangladesh

**Keywords:** Antioxidant, Antidiabetic, Extrusion technology, Functional foods, Mango peel, Noodle, Sensory properties

## Abstract

Antioxidant compounds such as phenolics and carotenoids scavenge reactive oxygen species and protect against degenerative diseases such as cancer and cardiovascular disease when used as food additives or supplements. Mango peel is a by-product of mango which is a good source of bioactive substances such as phytochemicals, antioxidants, and dietary fibers. Unfortunately, the study on mango peel as a potential food additive is very limited. Accordingly, the present study aimed to develop functional noodles through extrusion technology with the encapsulation of mango peel powder as a natural source of bioactive compounds. First, mango peel powder (MPP) was prepared and incorporated during the mixing of ingredients before noodles formation at three different levels (2.5, 5 and 7.5 %). Afterward, the noodles were studied to determine how the encapsulated MPP affects the proximate composition, physicochemical characteristics, polyphenols, carotenoids, anthocyanin, antioxidant and antidiabetic activity, and sensory characteristics. The noodles exhibited a dose-dependent relationship in the content of bioactive components and functional activities with the encapsulation of MPP levels. A significantly (p 0.05) higher value was noticed in 7.5 % of MPP-encapsulated noodles than in any level of MPP encapsulation in noodles. The fiber and protein contents in the MPP-encapsulated noodles were increased by about 0–1.22 % and 0–3.16 %, respectively. However, noodles’ color index and water absorption index were decreased with the level of MPP encapsulation. The cooking loss of noodles increased from 4.64 to 5.17, 6.49, and 7.32 %, whereas the cooked weight decreased from 35.11 to 34.40, 33.65, and 33.23 % with 2.5, 5.0, and 7.5 % of MPP encapsulation, respectively. However, MPP was stable during storage of noodles exhibiting higher phenolic content and antioxidant activity than control samples. The sensory evaluation showed that MPP-encapsulated noodles at levels 2.5 and 5 % had approximately similar overall acceptability values with the control sample. As a result of the findings, it appears that adding MPP up to 5 % to noodles improves their nutritional quality without changing their cooking, structural, or sensory aspects. Therefore, mango peel powder can be a potential cheap source for the development of functional noodles and food ingredients.

## 1 Introduction

Noodles are a popular staple food in many parts of the world such as China, Japan, Korea, Philippines, Indonesia, Vietnam, and Italy. It has been consumed for thousands of years (i.e. 4000 years ago in China) in many countries due to its ease of preparation, safety, appealing sensory quality, nutritional quality, extended shelf life, suitability, and affordable pricing [1]. Rathod and Annapure [2] reported that the consumption of noodles has become increasingly popular all over the world with changes in dietary habits being one of the main reasons for this trend. In many Asian diets, noodle is a major carbohydrate-based food and a substantial source of wheat products [2]. However, the increasing consumer demand for a healthy diet has promoted the research toward functional wheat-based noodle (i.e., rich in bioactive compounds, and dietary fiber) development. Therefore, noodles could be an excellent carrier for encapsulating nutrients like dietary fiber and bioactive compounds such as phenolics, anthocyanins, and carotenoids which are lacking in wheat flour. The global food industry diversifies the research into the invention and refinement of new and acceptable noodles that meet customer preferences is on the horizon [3]. Fruit processing byproducts such as peel, seed, and pomace have been discovered to be a rich source of phytochemicals that can be employed as functional food ingredients/additives [[4], [5], [6]]. So, the development of functional wheat-based noodles by encapsulating mango peel powder can be an alternative to meet the adequate intake of bioactive compounds in a human diet.

Mango (Mangifera indica L.) is a tropical fruit that is widely traded around the world [7]. During mango processing, peels are one of the major by-products of this fruit, accounting for 15–20% of the whole fruit [8]. Generally, peels are dumped as waste and become a source of pollution. Presently, peels are not utilized for any economic purposes in the food industries in Bangladesh. They are just treated as food waste by incineration, composting, landfill, or reutilizing for agricultural applications. As a result, these technologies allow to effectively minimize waste volumes but are not able to exploit the substantial potential of the waste as a crucial dietary source of phenolic compounds with potent antioxidant capacity, which could generate economic benefits. Mango peels have been demonstrated to be a good source of polyphenols, carotenoids, flavonoids, anthocyanins, vitamins, and other bioactive substances with a variety of health benefits [6,9,10]. Previous research has revealed that mango peels contain fiber that can be used as a food ingredient and that gives a variety of health benefits [[10], [11], [12]]. Due to their significant health-promoting properties, there is a growing interest in encapsulating mango peel powder into food products to develop functional foods like functional noodles. At the same time, the mango peel can be used to generate economic profit in the food industry as a source of nutrients and nutraceutical compounds, thereby partially mitigating and solving environmental problems [8,13].

Bioactive food components have the characteristic that they are subject to rapid inactivation or degradation. A novel strategy to increase the effectiveness and range of application of many types of natural functional ingredients is to use encapsulation delivery systems [14]. By encapsulation process many bioactive food components would therefore benefit from it that slows down the degradation processes, eventually preventing degradation until the product is delivered at the sites where adsorption is desired [15]. Among many encapsulation processes, the extrusion technique is promising for encapsulating bioactive compounds into foods considering their unique molecular characteristics [16]. The extrusion process is used worldwide for the production of noodles, as there is a huge demand for healthy and nutritious instant products from all age groups of consumers [17]. Therefore, the objective of this study was to develop functional noodles through extrusion technology with the encapsulation of mango peel powder as a natural source of bioactive compounds. Accordingly, the quality parameters such as cooking characteristics (color, cooking loss, cooked weight, and water absorption index), polyphenols, carotenoids, dietary fiber content, and antioxidant and antidiabetic activity of the developed noodles were also assessed.

## 2 Materials and methods

### 2.1 Preparation of mango peel

Fresh, disease-free, and ripe mangoes (*Mangifera indica* L.) *cv*. ashina were obtained from the local market of Dinajpur, Bangladesh. Whole mangoes were washed with tap and distilled water and peeled manually from the edible part of the fruit. The peels were sliced into small pieces and spread onto trays for hot-air cabinet drying at 60 ± 5 °C according to our previous study [18]. The dried mango peel was pounded using a grinder machine (Jaipan, JFM 1300). The pounded mango peels passed through a sieve (Mesh # 80) to get fine powder samples (particle size 0.18 mm) which were kept at −18 °C until further analyses and the preparation of value-added noodles.

### 2.2 Noodles formulation

The noodles were prepared by dry-mixing commercial wheat flour (WF) with different levels of mango peel powder (MPP) such as 0 % (control), 2.5 %, 5 %, and 7.5 %. Then, the premixed flour (500 g) was transferred to a single screw noodles maker (Model-HR2365/05, Philips, China) and warm (40 ± 2 °C) drinking water (150 mL) was added slowly and kneaded for 10 min for preparing the dough. Thereafter, the dough was made and the noodles maker automatically extruded (40 ± 2 °C) the dough through a die from which we obtained round-shaped rod noodles. These extruded round-shaped rod noodles were dried by a hot-air cabinet drier at 85 ± 5 °C °C for 3 h. Finally, dried noodles were cooled and packed (100 g) in polyethylene bags until further utilization, to prevent moisture and other contaminants.

### 2.3 Packaging and storage

The noodles were cut into a length of 20 cm, which were then packed and sealed into a low-density polyethylene bag to prevent moisture gain. The weight of each package was about 50 g and was stored in a cabinet at ambient conditions. Three packages were taken as a triplicate analysis of physical, chemical, cooking quality, sensory, and bioactive properties at the initial storage time for each group of samples. The noodles were stored up to 4 months for the analysis of the storage effect on total phenolic content and antioxidant activity of noodles and analyzed at 1-month intervals. According to test requirements, the noodle samples were ground by using a grinding machine (Jaipan, JFM 1300) and passed through a sieve (Mesh # 80) to get fine powder samples.

### 2.4 Analysis of proximate compositions

The oven drying method was used to determine the moisture content, and the ash content was measured by dry combustion using the temperature of 550 °C to 600 °C for 5–6 h or until a constant weight was reached, an acid-alkaline digestion method was used to determine fiber content and crude fat was determined by using petroleum ether extraction according to AOAC method 7.045. Protein was determined by using the Lowry method [19] which is based on the biuret reaction. The total carbohydrate content of samples was calculated by subtracting the value of moisture, ash, fat, fiber, and protein from 100 as follows:

%Carbohydrate=100–(moisture%+ash%+fat%+fiber%+protein%)

### 2.5 Evaluation of noodles quality

#### 2.5.1 Noodles cooking properties

The cooking quality of the noodles was determined in terms of cooking loss and WAI (water absorption index). Cooking loss was evaluated according to the method described by Ref. [11]. Briefly, 10 g of dry noodles was put into 200 mL of boiling water and cooked for 10 min with occasional stirring. By using a Buchner funnel, the gruel was drained and rinsed with 50 mL of drinking water at room temperature for 30 s and endorsed to exude for 2 min. Collect the gruel and rinsed water, then the volume (v) was measured. For even distribution of the solid content, collected gruel was properly shaken and 20 mL of the aliquot was taken into a Petri dish and subjected to oven drying at 105 ± 2 °C until a constant weight was attained and the cooking loss (%) was determined. The water absorption index (WAI) was calculated by weighing noodles before and after cooking [20]. The ratio percentage increment of noodles weight during cooking was expressed as WAI.

#### 2.5.2 Color measurement of the noodles

The cooked noodle's color was measured by a colorimeter (Biobase, China) using specular reflectance induced with D65 illuminant and an 8° observer angle. Cooked noodles (10 g) were placed on a transparent glass plate and the noodle's surface color was measured three times by a colorimeter. The average value of three measurements was reported for L* a* b* values, where L* indicates whiteness/darkness, a* represents redness/greenness (positive value = redness, negative value = greenness), and b* represents yellowness/blueness (positive = yellowness, negative = blueness). By using L* and b* values, the color of the noodles index (NCI) was determined using the following equation #1 [21].

(1) $$\text{NCI}=\sqrt{L^{*2}+a^{*2}}$$

#### 2.5.3 Sensory analysis

For sensory evaluation, noodles were cut into 6 cm pieces, and cooked in boiling water for 10 min, and exuded as described earlier. The samples were then transferred to the glass plate and left for not more than 5 min before sensory evaluation. A total of 35 expert panelists comprising teachers, students, and staff of the Food Processing and Preservation Department, HSTU, Bangladesh were selected to evaluate the quality attributes of the cooked noodles. The panelists were asked to rate their liking of the noodles by color/appearance, discreteness, firmness/stickiness, taste, flavor, and overall quality using a 9-point hedonic scale of 1–9 (9 = like extremely and 1 = dislike extremely).

#### 2.5.4 Extraction of bioactive compounds

All dried noodles samples were pounded with a mortar and pestle. The pounded samples were extracted for bioactive compounds by using organic solvent by the protocol of [18] with some modifications. The pounded samples (2.5 g) were mixed with 50 mL of 80 % methanol in a glass conical flux with a solid/liquid ratio of 1:20 (g/mL) and extracted at room temperature for 60 min in a shaking water bath at 100 rpm. Subsequently, the sample was centrifuged (general centrifuge MF-300, HumanLab Instrument Co., Korea) at 4000 rpm for 10 min. Then, the supernatant was transferred and filtered through a Whatman no 1 filter paper before the analysis.

##### 2.5.4.1 Estimation of total phenolic content

The total phenolic content of noodle samples was determined according to the Folin-Ciocalteu method described by Hasan et al. [22]with some modifications. First, 0.5 mL of the sample was taken in a centrifuge tube and 0.5 mL of Folin-Ciocalteu reagent was added to it. The contents were mixed thoroughly and 1 mL of saturated sodium bicarbonate (7.5 % solution) was added to each tube for neutralization. Afterward, 8 mL of distilled water was added and vortexed thoroughly. Then, the mixtures were centrifuged for 10 min at 4000 rpm and allowed to stand for 35 min at room temperature in the dark. The absorbance of the resultant black-colored supernatant was read at 750 nm using a spectrophotometer (UV-1900i, Shimadzu, Japan) and an appropriate blank was used for background subtraction. Then, the TPC was estimated and expressed as milligrams of gallic acid equivalents per gram of dry matter (mg GAE/g DM).

##### 2.5.4.2 Estimation of total flavonoid content

The total flavonoid content of the noodles was determined using the AlCl_3_ (Aluminium chloride) colorimetric method with some modifications as described by Ref. [6]. Firstly, 1 mL of the extracted sample was mixed with 4 mL of distilled water and 0.3 mL of 5 % NaNO_2_ in falcon tubes. The tubes were then allowed to stand for 5 min and subsequently, 0.3 mL of 10 % AlCl_3_ was added to the mixture and allowed once again to stand for 1 min. Finally, 2 mL of 1 M NaOH and 2.4 mL of distilled water were added and mixed immediately. The absorbance of the mixture was read at 510 nm after 15 min of incubation followed by centrifugation at 4000*g* for 5 min against a blank prepared manner similarly. The results were expressed as mg quercetin equivalents per gram of dry matter (mg QE/g DM) calculated from a standard curve of quercetin.

##### 2.5.4.3 Total anthocyanin content

A spectrophotometric pH differential procedure was used to determine the total anthocyanin content of samples [23]. To enhance the detection limit, 300 mg of dried sample was extracted with 2 mL of various solvents. Then 1 mL extracts were well mixed with 0.025 M potassium chloride pH 1.0 buffer in a 1:36 extract-to-buffer ratio, and the absorbance of the mixture was measured at 510 and 700 nm after 15 min of reaction. Again, the extracts were mixed in the same ratio with a 0.4 M sodium acetate buffer pH 4.5, and the absorbance of the mixture was measured at the same wavelengths. The content of anthocyanin in the extracts was estimated by equation #2:

(2) $$\text{The}\;\text{total}\;\text{anthocyanin}\;\left(\frac{\mu g}{L}\right)=\frac{\left(\left(A_{510}-\;A_{700}\right)pH\;1-\left(A_{510}-\;A_{700}\right)pH\;4.5\right)\times MW\times 1000}{\varepsilon \times l}$$

where *A* is absorbance, *MW* is the molecular weight for cyanidin 3-glucoside (449.2 g/mol), Ɛ is the molar extinction coefficient of cyanidin 3-glucoside (26,900 L mol^−1^ cm^−1^) and *l* is path-length (cm), 1000 is the factor for conversion g to μg and expressed as μg of cyanidin 3-glucoside equivalents/100 g sample DM.

##### 2.5.4.4 Estimation of total carotenoid

The total carotenoid content was measured according to the procedure of Hasan et al. [24]. In brief, 5 g of samples were mashed by mortar and pestle and dissolved in 50 mL of n-hexane-acetone-ethanol solution (at a ratio of 50:25:25) (v/v) in a conical flask. Then, the extraction of carotenoids was performed in a shaking water bath at 100 rpm for 10 min at room temperature followed by centrifugation (General centrifuge MF – 300, HumanLab Instrument Co., Korea) at 6500 rpm for 5 min at 4 ͦ C. The absorbance was recorded at 450 nm by using a spectrophotometer (UV-1900i, Shimadzu Scientific Instruments Inc., Japan) and the results were expressed as μM β-carotene equivalent per gram sample using β-carotene as standard.

##### 2.5.4.5 Estimation of antioxidant activity

Two antioxidant assays were performed to evaluate the antioxidant activities of the noodles. First, the DPPH (2, 2-diphenyl-1-picrylhydrazyl) scavenging ability assay was performed using the protocol of Islam et al. [6]. A solution of DPPH in 80 % methanol was stirred for 30 min and the absorbance of the solution was adjusted to 0.650 at 515 nm using a spectrophotometer (UV-1900i, Shimadzu, Japan). Then, the mixture of 50 μL of the extracted sample and 1.950 mL of DPPH was vortexed and left in the dark for 30 min at room temperature. After that, the absorbance of the mixture was measured at 515 nm and the results were expressed as μM Trolox equivalents per gram of dry matter (μM Trolox/g DM) calculated from a standard curve of Trolox.

Second, the ferric-reducing antioxidant power (FRAP) test was carried out according to the reference of [25] with slight modification. The absorbance of the reaction mixture of 25 μL extracted samples and 1.975 mL of the FRAP reagent (prepared by mixing 20 mM iron (III) chloride solution, 10 mM TPTZ (2,4,6-Tris(2-pyridyl)-*s*-triazine) solution and 40 mM HCl in 10:1:1 (v/v) ratio using acetate buffer (pH 3.6)) was recorded at 593 nm after 4 min of incubation. The results were expressed as μM Fe (II) Equivalents/g DM, respectively using iron (II) sulfate as standard.

##### 2.5.4.6 Analysis of antidiabetic activity

The α-glucosidase inhibitory activity was measured by the protocol of [18] to examine the antidiabetic activity of noodles. In detail, 50 μL of the extracted sample was mixed with 100 μL of 0.1 U/mL α-glucosidase solution (in 0.1 M, pH 6.9 phosphate buffer). After 10 min of reaction at 25 °C, 50 μL of 5 mM *p*-Nitrophenyl-α-d-glucopyranoside solution (in 0.1 M, pH 6.9 phosphate buffer) was added and the mixture was allowed to incubate at 25 °C for 5 min. Then, the absorbance was measured at 405 nm using a spectrophotometer, and the result was expressed as μM Acarbose equivalents per gram of dry sample (μM Acarbose/g DM) using Acarbose as standard.

### 2.6 Statistical analysis

All analyses were performed in triplicate and all of the results were presented as means ± standard deviation. Statistical analysis was performed (SPSS software, version 26.0) by one-way analysis of variances (ANOVA). Mean comparisons were performed using Duncan's multiple range tests for significant effect at p ≤ 0.05.

## 3 Results and discussion

### 3.1 Proximate compositions

The proximate compositions of the six samples such as wheat flour (WF), mango peel powder (MPP), and noodles with 0 % (control), 2.5 %, 5.0 %, and 7.5 % of MPP were presented in Table 1. The results have shown that the moisture content did not differ significantly (p > 0.05) among all samples. However, the MPP exhibited the highest amount of ash (3.84 %) while the lowest was in WF (0.90 %). Consequently, the encapsulation of MPP in noodles contributed significantly (p > 0.05) to the ash content of 3.5 %, 3.1 %, and 2.9 % with 7.5 %, 5.0 %, and 2.5 % of MPP, respectively. The ash content can be credited to the number of minerals present in the sample [26]; therefore, the addition of MPP in the developed noodles might increase the number of minerals. The total protein content in the samples ranged from 9.05 % to 13.66 %. The protein content of WF and MPP is an important issue because it influences the cooking quality of noodle products [27]. Furthermore, protein is an excellent source of amino acids for humans. It is well known that amino acids are important for metabolic health, tissue repair, and the synthesis of hormones and neurotransmitters [28,29]. Hence, the encapsulation of MPP can increase the protein content of the developed noodles. Expectedly, the study noticed significantly (p < 0.05) higher protein content in the noodles developed with 7.5 % MPP than 5.0 %, 2.5 %, and 0 % (control) of MPP. The fat content of the noodles encapsulated with MPP was slightly higher than the control. It could be due to the higher amount of fat in MPP (2.1 %) than in WF (1.84 %). From a nutritional point of view, high fat in noodles is good, and oil from mango peel could be a high-quality edible plant oil that may promote our health due to its fatty acid compositions and phenolic compounds [30]. However, it could be undesirable because the fat rancidifies during storage and leads to a displeasing taste and odor to the finished product [31]. Notably, the MPP had the highest amount of crude fiber (18.33 %), as expected, the encapsulation of MPP in noodle formulation significantly (p < 0.05) increased the amount of fiber (%) in noodles supplemented with 7.5 % MPP (3.32) followed by 5.0 % MPP (2.91), 2.5 % MPP (2.51) and control (2.10). Several studies have shown that fiber intake is essential for modulating and supporting gut microbiota [32]. Fiber consumption has also been linked to a lower risk of colorectal cancer, diabetes, and inflammation [33]. As a result, noodles partially substituted with MPP represent a good healthy option for fiber-rich diets. The carbohydrate content (%) of the noodles encapsulated with MPP was significantly (p < 0.05) lower than the control (74.18) where 7.5 % MPP supplementation showed the lowest amount of carbohydrate (66.92) followed by 5.0 % (70.61) and 2.5 % MPP (71.81). The encapsulation of MPP in noodles provides a moderate amount of carbohydrates in this study. Therefore, people with diabetes can enjoy noodles encapsulated with MPP in their diets for potential health benefits [34]. reported that the total amount of carbohydrates in meals is more important than the source and type of carbohydrates for the person with diabetes in regards to the glycemic effects of carbohydrates.

**Table 1:** Proximate composition (g/100 g dry sample).

Sample	Moisture (%)	Ash	Protein	Fat	Fiber	Carbohydrate
**WF**	10.79 ± 0.08^a^	0.587 ± 0.02^f^	10.19 ± 0.09^d^	1.84 ± 0.06^d^	1.98 ± 0.33^e^	74.61 ± 3.31^a^
**MPP**	10.65 ± 0.06^a^	3.84 ± 0.04^a^	9.05 ± 0.08^e^	2.10 ± 0.53^a^	18.33 ± 1.26^a^	56.03 ± 4.43^d^
**Control**	10.44 ± 0.04^a^	0.9 ± 0.07^e^	10.50 ± 1.04^c^	1.88 ± 0.45^c^	2.10 ± 0.04^e^	74.18 ± 5.23^a^
**S1**	10.36 ± 0.07^a^	2.9 ± 0.05^d^	10.52 ± 1.03^c^	1.90 ± 0.57^bc^	2.51 ± 0.10^d^	71.81 ± 3.29^b^
**S2**	10.55 ± 0.05^a^	3.1 ± 0.04^c^	10.90 ± 1.05^b^	1.93 ± 0.36^bc^	2.91 ± .09^c^	70.61 ± 3.37^bc^
**S3**	10.63 ± 0.03^a^	3.5 ± 0.06^b^	13.66 ± 1.08^a^	1.97 ± 0.41^b^	3.32 ± 0.11^b^	66.92 ± 3.17^c^

Each value is expressed as mean ± SD and different small letters within the same column indicate significant (p ≤ 0.05) differences among various samples. Note: WF: wheat flour, MPP: mango peel powder, Control - denotes noodles with unencapsulated MPP, and S1, S2, and S3 denote noodles encapsulated with 2.5, 5.0, and 7.5 % of MPP.

### 3.2 Physicochemical characteristics

The physicochemical properties of the noodles such as color, water absorption index, and cooking quality were evaluated and presented in Table 2. The instrumental color measurements are much more accurate than visual analysis and provide a data system for dark, white, or any other colors. The color of the food products is an important parameter for consumers that influences food sales. Accordingly, the colors of noodles were measured by calorimeter in terms of L*, a*, and b* values. The L* values (whiteness) were dropped from 42.67 to 22.08 (cooked noodles) and 58.75 to 38.08 (uncooked noodles) with the increasing level of MPP in the noodles compared to the control (Table 2). The a* and b* values were increased significantly (p < 0.05) to about the MPP content of both uncooked and cooked noodles. MPP levels in noodles also affect the Noodle Color Index (NCI). When the MPP level is increased, the NCI value significantly (p < 0.05) dropped from 43.00 to 23.50 (cooked noodles) and 60.63 to 42.86 (Uncooked noodles). The encapsulation of MPP is mostly responsible for the lowering of whiteness (ΔL <0), and for the rise in redness (Δa>0) and yellowness (Δb>0). Moreover, the development of darkness, redness, and yellowness in the cooked noodles might be due to the Maillard reaction between reducing sugars and proteins during cooking [35].

**Table 2:** Physiochemical characteristics of MPP extruded and control noodles.

Sample	L*	a*	b*	Noodles Color Index	Water Absorption Index (%)	Cooking loss (%)	Cooked Weight (g)
Cooked Noodles							
**Control**	42.67 ± 1.23^a^	2.72 ± 0.44^c^	5.33 ± 0.15^c^	43.00 ± 1.22^a^	250.23 ± 0.98^a^	4.64 ± 0.32^d^	35.11 ± 0.51^a^
**S1**	29.59 ± 1.44^b^	5.3 ± 1.15^b^	5.75 ± 0.06^c^	30.15 ± 1.41^b^	243.58 ± 0.20^b^	5.17 ± 0.38^c^	34.40 ± 0.4^7a^
**S2**	25.38 ± 0.69^c^	6.93 ± 0.40^a^	6.80 ± 0.41^b^	26.27 ± 0.77^c^	235.6 ± 0.65^c^	6.49 ± 0.36^b^	33.65 ± 0.49^ab^
**S3**	22.08 ± 1.73^d^	8.00 ± 0.35^a^	8.00 ± 0.40^a^	23.50 ± 1.63^d^	231.67 ± 0.21^d^	7.32 ± 0.33^a^	33.23 ± 0.61^ab^
**Fresh Uncooked Noodles**							
**Control**	58.75 ± 0.42^a^	4.00 ± 0.64^c^	15.01 ± 0.54^c^	60.63 ± 0.34^a^	–	–	–
**S1**	48.67 ± 0.65^b^	7.39 ± 0.17^b^	17.12 ± 0.86^b^	51.6 ± 0.33^b^	–	–	–
**S2**	44.59 ± 0.68^c^	7.52 ± 0.59^ab^	17.34 ± 0.40^b^	47.84 ± 0.59^c^	–	–	–
**S3**	38.08 ± 0.81^d^	8.30 ± 0.20^a^	19.67 ± 0.96^a^	42.86 ± 1.14^d^	–	–	–

Each value is expressed as mean ± SD and different small letters within the same column indicate significant (p ≤ 0.05) differences among various samples. Note: Control-denotes noodles with unencapsulated MPP, and S1, S2, and S3 denote noodles encapsulated with 2.5, 5.0, and 7.5 % of MPP.

The water absorption index (WAI) of the noodles was decreased with increased levels of MPP encapsulation (Table 2). After cooking, the high WAI was noticed in the control (its weight increased 2.50 times) followed by noodles encapsulated with 2.5 % (2.43 times) 5.0 % (2.35 times), and 7.5 % MPP (2.31 times), respectively. Many factors can explain the decrease in the WAI of the MPP-enriched noodles. For instance, a higher protein network might be formed in MPP-enriched noodles that prevents the diffusion of water into the starch granules and prevents their swelling [36]. However, The WAI of the noodles was similar to those reported by the other authors in pasta [20] and spaghetti-type pasta [37].

The results obtained for the cooking quality (weight and cooking loss) of the noodles enriched with MPP are shown in Table 2. The noodles encapsulated with MPP have a lower cooked weight than the control noodles. The cooked weight was decreased with the increasing levels of MPP in the noodles. The cooking loss is measured as an indicator of the cooking process based on the amount of solid leached in the cooking water. The noodles enriched with MPP showed significantly (p < 0.05) higher cooking loss than the control sample. The reason for the increase in cooking loss of the encapsulated noodles may be due to several factors including changes in the starch and fiber content, as well as alterations in the water-holding capacity and gluten-protein network in the noodles. For instance, Kim et al. [38] reported that the fiber incorporation in pasta increased cooking loss by influencing the diffusion of cooking water and weakening gluten networks. Moreover, the high fiber content in MPP might disrupt the gluten-protein network [11], and protein-starch matrix by the uneven distribution of water due to the competitive hydration tendency of the fiber [39], thus resulting in a high cooking loss in MPP-encapsulated noodles [40]. noted that the decrease in the cooked weight of the noodles was due to the increase in the cooking loss which was in agreement with this study [41]. reported that a cooking loss of 12 % or less is considered indicative of good-quality pasta. Therefore, the noodles encapsulated with MPP (all levels) can be considered good quality noodles since they represented a cooking loss of 5.17–7.32 %.

### 3.3 Sensory evaluation of noodles

**Fig. 1:**
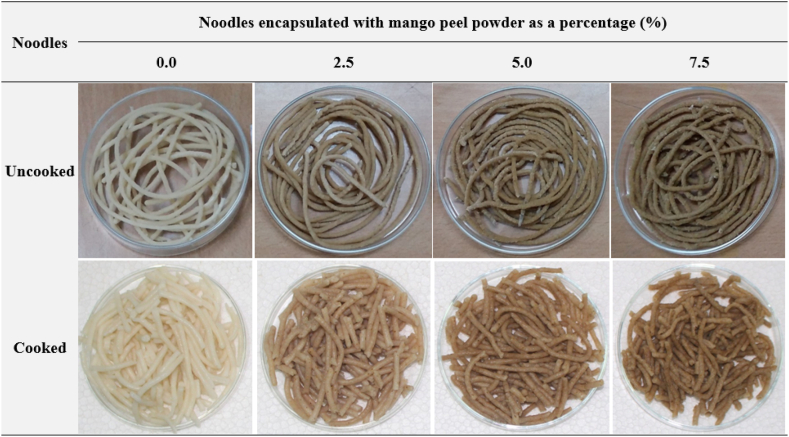
Photograph of noodles inclusion with mango peel powder.

**Fig. 2:**
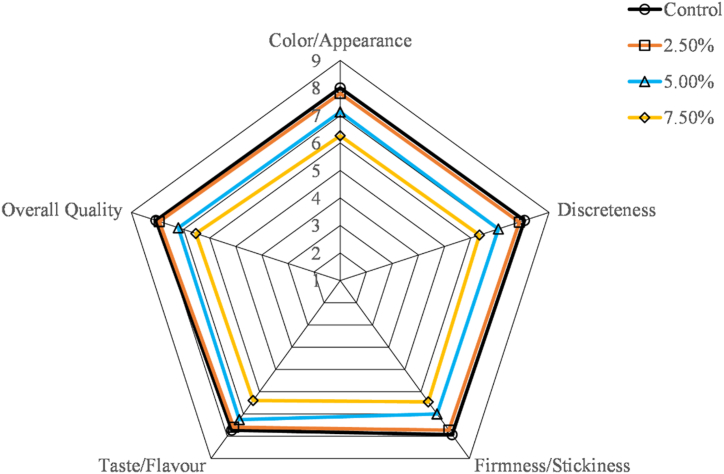
Influence of mango peel powder on the sensory acceptability of noodles. Note: Control-denotes noodles with unencapsulated MPP, and 2.5, 5.0, and 7.5 denote noodles encapsulated with 2.5, 5.0, and 7.5 % of MPP.

The results of the 9-point hedonic test on sensory parameters such as color/appearance, discreteness, firmness/stickiness, taste/flavor, and overall quality of the cooked noodles encapsulated with different levels of MPP were presented in Fig. 2. The encapsulation of MPP at different levels had considerable effects on the noodle's sensory quality. Noodles prepared with the encapsulation of 2.5 % and 5 % MPP had a yellowish creamy hue and a yellow color, respectively while the control noodles had a white color (Fig. 1). The color of noodles made with 7.5 % MPP was yellowish brown. The yellowish appearance of the noodles might be due to the addition of MPP and its carotenoid pigment contents [42]. The hedonic scores obtained for color/appearance, firmness/stickiness, and taste/flavor varied from 7.8 to 7.13, 7.73 to 7.0, and 7.6 to 7.26, respectively while for control it was almost 8.0; there was no significant (p > 0.05) difference between control noodles (100 % WF) and MPP encapsulated noodles (up to 5 % MPP). However, noodles with 7.5 % MPP had a lower score on taste and flavor, as well as an unappealing appearance to the panelist. The encapsulated noodles scored similarly in terms of discreteness and firmness due to hard and non-sticky characteristics except for 7.5 % MPP. The cooking loss of the noodles had the greatest impact on the sensory qualities [43]. [44] reported that the maximum cooking loss causes turbidity in cooking water; thereby decreasing cooking tolerance and mouth feel. Based on the sensory tests, it was estimated that MPP may be used up to 5 % in the noodle formulation without touching the sensory quality. This statement might be consistent with the findings of the proximate analysis of noodles.

### 3.4 Bioactive compounds in MPP and noodles

Encapsulation of MPP as an ingredient in noodle formulation significantly (p < 0.05) increased the content of total phenolics, flavonoids, carotenoids, and anthocyanin in comparison to control noodles (Table 3). The inclusion of MPP in noodles raised the total phenolic content (TPC) from 1.33 to 9.63 mg GAE/g noodles sample, thus resulting in 7.2 times higher than the control. The total flavonoid content (TFC) of the noodles incorporated with different levels of MPP was significantly (p < 0.05) higher than control noodles. The highest TFC was noticed in 7.5 % MPP encapsulated noodles (5.17 μg QE/g) whereas the lowest was in the control sample (0.58 μg QE/g). The incorporation of MPP from 0 to 7.5 % enhanced (p < 0.05) the carotenoid content in the noodles from 2.27 to 318.29 μM/g noodles. The anthocyanin content also significantly (p < 0.05) increased from 0.83 to 23.12 μg cyanidin 3-glucosidise equivalents/100 g with the addition of MPP from 0 to 7.5 % in the noodles. The rate of the increase of the content of bioactive compounds such as TPC, TFC, carotenoid, and anthocyanin was proportional to the concentration of MPP in the noodles in this study. Similar results were also noticed by other authors with the addition of apple flour in pasta [20], pomelo fruit segment in noodles ([45], and mango peel powder in macaroni [11]. Moreover, the high content of bioactive compounds also was apparently due to the high content of total phenolic (26.30 mg GAE/g DM), TFC (13.48 μg QE/100 g), carotenoids (1171.65 μM/g) and anthocyanin (33.69 μg cyanidin 3-glucosidise equivalents/100 g) in MPP [6,11]. This natural bioactive compound acts against many diseases like diabetes, cancer, and other degenerative diseases [11,46]. Anthocyanin has been known for its functional role against cancers, diabetes, and cardiovascular and neurological diseases [47,48]. Therefore, encapsulating these compounds as natural ingredients is suggested to enhance the functionality of value-added food products.

**Table 3:** Bioactive components (DPPH, FRAP, TPC, TFC, Carotenoid, α-glucosidase, and Anthocyanin) of MPP, MPP incorporated and control noodles.

Sample	TPC (mg GAE/g DM)	TFC μg QE/g DM	Carotenoid μM β-carotene/g	Anthocyanin μg cyanidin 3-glucosidase equivalents/100 g	DPPH μM Trolox/g DM	FRAP μM Fe (II)/g DM	α-glucosidase μM Acarbose/g DM
**Control**	1.33 ± 0.05^e^	0.58 ± 0.13^e^	2.27 ± 1.54^e^	8.36 ± 0.16^e^	32.87 ± 1.96^e^	38.64 ± 2.69^e^	2.75 ± 0.11^e^
**S1**	5.54 ± 0.03^d^	1.36 ± 0.11^d^	108.99 ± 1.08^d^	14.09 ± 0.08^d^	208.91 ± 1.36^d^	335.25 ± 3.26^d^	4.59 ± 0.08^d^
**S2**	7.52 ± 0.04^c^	4.09 ± 0.17^c^	227.02 ± 0.89^c^	19.25 ± 0.10^c^	264.24 ± 0.65^c^	495.68 ± 3.26^c^	6.69 ± 0.18^c^
**S3**	9.63 ± 0.15^b^	5.17 ± 0.15^b^	318.29 ± 1.31^b^	23.12 ± 0.08^b^	335.05 ± 2.47^b^	621.17 ± 4.27^b^	8.22 ± 0.16^b^
**MPP**	26.30 ± 1.18^a^	13.48 ± 0.12^a^	1171.65 ± 6.02^a^	33.69 ± 0.62^a^	359.23 ± 0.99^a^	2664.31 ± 4.32^a^	10.66 ± 0.06^a^

Each value is expressed as mean ± SD and different small letters within the same column indicate significant (p ≤ 0.05) differences among various samples. Note: Control-denotes noodles with unencapsulated MPP, and S1, S2, and S3 denote noodles encapsulated with 2.5, 5.0, and 7.5 % of MPP.

### 3.5 Antioxidant and antidiabetic activities in MPP and noodles

The DPPH and FRAP methods were used to determine the antioxidant capacity of MPP and encapsulated noodles products. The results in Table 3 showed that MPP-incorporated noodles had significantly (p < 0.05) higher antioxidant capacity both in DPPH and FRAP assays than control noodles and ranked as follows: noodles with MPP of 7.5 % > 5.0 %>2.5 % > 0 % (control). In this regard, the scientific literature reports that the mango peel has a high content of different bioactive compounds such as carotenoids, phenolic acids, flavonoids, and vitamins C and E [6,42] which are directly related to the greater antioxidant capacity of MPP (Table 3). Thus, the encapsulation of MPP in noodles greatly increased their antioxidant activities. Antioxidant scavenges free radicals [49], and contributes to the antioxidative defense mechanism in living cells by terminating the chain reaction that protects human health. Therefore, MPP-encapsulated noodles can provide the benefits of antioxidants that inhibit different generative diseases in the human body. Similar results were also noticed in pasta, noodles, and macaroni formulated with apple flour [20], pomelo fruits [45], and mango peels [11], respectively.

The α-glucosidase is a key enzyme that digests carbohydrates to covert glucose and leads to an increased level of postprandial glucose [50]. noticed that polyphenol compounds have antidiabetic outcomes by inhibiting the activity of α-glucosidase. In this study, MPP as a source of polyphenol compounds (Table 3) was encapsulated into noodles. To investigate the antidiabetic activity of MPP-encapsulated noodles, the α-glucosidase inhibition activity was performed and shown in Table 3. The MPP incorporated in all noodles showed significantly (p < 0.05) higher α-glucosidase inhibitory activity than control noodles. The addition of the amount of MPP in noodles was directly proportional to the inhibition activity of the enzyme. High inhibition was noticed in the MPP-encapsulated noodles due to the high inhibition capacity of MPP [11]. Therefore, the findings are in agreement with several reports demonstrating that mango peel or fruit byproducts or underutilized edible plants possess α-glucosidase inhibitory activity [51]. Therefore, MPP-encapsulated noodles might be considered functional foods to control diseases like diabetes.

### 3.6 Effect of storage time on total phenolic content (TPC) and antioxidant activity in noodles

**Fig. 3:**
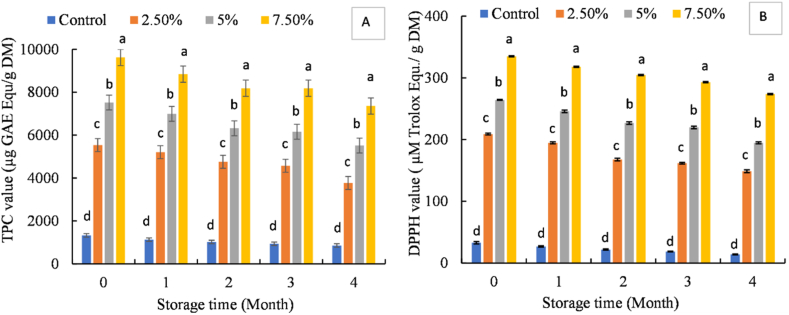
TPC (A) and DPPH radical scavenging ability (B) of the noodles during storage. Each value is expressed as mean ± SD and different small letters within the same column indicate significant (p ≤ 0.05) differences among various samples.

Phenolic compounds are one of the most important groups of phytochemicals that exert health benefits to the human body by scavenging the free radicals which may induce a series of diseases such as arthritis, cancer, diabetes, atherosclerosis, immune deficiency, and endocrine dysfunction [[52], [53], [54]]. Accordingly, the effect of storage time on the TPC and antioxidant activity of MPP encapsulated noodles at the ambient condition was evaluated and shown in Fig. 3(A) and (B). The results demonstrated that the TPC and antioxidant activity in terms of DPPH radical scavenging activity was raised with the increasing quantity of MPP during the initial period. The highest TPC and antioxidant activity were observed in the noodles sample with the addition of any level of MPP, which was significantly (p < 0.05) higher than the noodles without MPP (control sample). However, the gradual decrease of TPC and antioxidant activity was observed with the increase in storage time in all noodles. Nevertheless, it demonstrated that MPP was a stable ingredient in noodles, and the content of phenolic compounds and antioxidant activity were maintained over the storage time with a slight decrease. Studies have shown that during noodle processing, the presence of water, oxygen, heat, and suitable environment-activated polyphenol oxidase present in flour may induce the oxidative degradation of phenolic compounds [54]. That may be the reason for decreasing the phenolic content in noodles. The decrease in antioxidant activity also depends on the decrease in total phenolic content, and the noodle's processing and storage time have a negative effect on antioxidant properties reported by Ref. [54].

## 4 Conclusion

Mango peel powder (MPP) has been discovered to be a potential food ingredient for the development of functional noodles in this investigation. This study reports that MPP contains a significant amount of phytochemicals like polyphenols, carotenoids, anthocyanins, and dietary fibers. Encapsulation of MPP enhanced the polyphenol, carotenoid, anthocyanins, and dietary fiber content of noodles while also improving their antioxidant and antidiabetic activity. The noodles mixed with MPP up to 5 % levels resulted in products with good acceptability with negligible comparison to control, according to investigations on physiochemical characteristics, and sensory assessments. Therefore, noodles prepared with MPP can be considered functional noodles with bioactive compounds which may be a convenient food format to satisfy consumer interest. During storage, the content of total phenolic (TPC) and antioxidant activity was stable in the MPP encapsulated for up to 3 months and then slightly decreased. Nevertheless, further research is needed to specify antioxidants and polyphenols by using HPLC analysis. Hence noodles enriched with MPP act as a good source of bioactive components and fiber that encourage a wider utilization of the MPP in the human diet for potential health benefits.

## Ethics statement

All relevant rules, guidelines, regulations were followed, and consent was sought and obtained from all panelists in this study.

## Data share statement

Data will be made available on request.

## CRediT authorship contribution statement

**Md Raihan Kabir:** Writing – original draft, Methodology, Investigation, Conceptualization. **S.M. Kamrul Hasan:** Writing – review & editing, Visualization, Supervision, Project administration, Funding acquisition, Conceptualization. **Md Rakibul Islam:** Writing – original draft, Data curation. **Maruf Ahmed:** Supervision.

## Declaration of competing interest

The authors declare that they have no known competing financial interests or personal relationships that could have appeared to influence the work reported in this paper.
