# Supplementary material for: Development of functional noodles by encapsulating mango peel powder as a source of bioactive compounds
Source: Heliyon. 2024 Jan 4;10(1):e24061. doi: 10.1016/j.heliyon.2024.e24061 (PMC10789624; doi:10.1016/j.heliyon.2024.e24061)
Supplement: Multimedia component 1 [file mmc1.docx]

**Questionnaire**

**Sensory evaluation of the developed food product (noodles)**

Name of the evaluator:

Designation/Occupation:

Age:

| Name of Parameter | | Appearance/Color | Discreteness | Firmness/  Stickiness | Taste/ Flavor | Overall Acceptability |
| --- | --- | --- | --- | --- | --- | --- |
| Sample | S1 |  |  |  |  |  |
|  | S2 |  |  |  |  |  |
|  | S3 |  |  |  |  |  |
|  | S4 |  |  |  |  |  |

The panelists were asked to rate their liking of the noodles by color/appearance, discreteness, firmness/ stickiness, taste, flavor, and overall quality using a 9-point hedonic scale.

Dislike extremely=1, Dislike very much=2, Dislike moderately=3, Dislike slightly=4, Neither like or dislike=5, Like slightly=6, Like moderately=7, Like very much=8, Like extremely=9

Signature of the evaluator
